# Ultradense and planarized antireflective vertical silicon nanowire array using a bottom-up technique

**DOI:** 10.1186/1556-276X-8-123

**Published:** 2013-03-09

**Authors:** Ludovic Dupré, Thérèse Gorisse, Angélique Letrouit Lebranchu, Thomas Bernardin, Pascal Gentile, Hubert Renevier, Denis Buttard

**Affiliations:** 1 SiNaPS Laboratory SP2M, UMR-E CEA/UJF-Grenoble 1, CEA/INAC, 17 Avenue des Martyrs, Grenoble 38054, France; 2 CNRS/LTM, 17 Avenue des Martyrs, Grenoble 38054, France; 3 CEA/INAC/SP2M/Lemma, 17 Avenue des Martyrs, Grenoble 38054, France; 4 LMGP, Grenoble INP-Minatec, 3 Parvis Louis Néel, Grenoble 38016, France; 5 UJF/IUT-1, 17 Quai Claude Bernard, Grenoble 38000, France

**Keywords:** Silicon nanowires, Vapor–liquid-solid, High density, Nanoporous alumina, Universal substrate, Light trapping

## Abstract

The production and characterization of ultradense, planarized, and organized silicon nanowire arrays with good crystalline and optical properties are reported. First, alumina templates are used to grow silicon nanowires whose height, diameter, and density are easily controlled by adjusting the structural parameters of the template. Then, post-processing using standard microelectronic techniques enables the production of high-density silicon nanowire matrices featuring a remarkably flat overall surface. Different geometries are then possible for various applications. Structural analysis using synchrotron X-ray diffraction reveals the good crystallinity of the nanowires and their long-range periodicity resulting from their high-density organization. Transmission electron microscopy also shows that the nanowires can grow on nonpreferential substrate, enabling the use of this technique with universal substrates. The good geometry control of the array also results in a strong optical absorption which is interesting for their use in nanowire-based optical sensors or similar devices.

## Background

Semiconductor nanowires are now widely implemented as active elements in devices for various applications such as energy harvesting [1,2], microelectronics [3], or sensors [4,5]. In order to achieve high performances, high densities of nanowires are required to increase efficiency or sensitivity of devices [6,7]. In this purpose, top-down etching of a semiconductor wafer is the most commonly used technique [7-9]. However, the requirement of a bulk wafer prevents the realization of cost-effective devices. Some groups therefore choose to use bottom-up techniques and produce nanowires using catalytic processes such as chemical vapor deposition (CVD) [10-12], allowing the growth of nanowires on noncrystalline substrates [13,14]. However, the production of high-density arrays of aligned nanowires is challenging with this technique because it requires a control of the density and localization of the metallic catalyst seeds. Furthermore, if the substrate is not oriented in the preferential growth direction, it is impossible to achieve arrays with aligned nanowires because of their random orientations on the substrate. Various solutions are investigated to create high-density networks of nanowires using a bottom-up approach. For instance, dense networks of gold droplets can be realized by dewetting a thin layer of gold deposited on the surface of a substrate [15], but the density is not as high as with top-down techniques, and the size of the catalyst particles is hardly controlled. Another interesting solution is to lithographically pattern a substrate with catalyst particles [16,17], which is time and money consuming in the case of e-beam lithography to achieve nanoscale dimensions.

We describe a new bottom-up method to produce silicon nanowire arrays which present a very high density and height homogeneity. Nanowires are grown by gold-catalyzed CVD in the vapor–liquid-solid (VLS) mode using an anodic aluminum oxide (AAO) membrane with cylindrical nanopores as growth template. This guided nanowire growth is used to create arrays of vertically aligned nanowires with densities up to 10^10^ cm^−2^ on substrates oriented in another direction than the preferential one [18,19]. It is usually admitted that in this case, growth has to be stopped before the nanowires reach the surface of the AAO template. It indeed prevents any structural anomalies such as kinks and increases of the nanowires’ diameter due to the catalyst getting out of the template. This leads to a difficult control and inhomogeneities in the length of the nanowires depending on the size of the initial gold catalyst. However, a planarized silicon nanowire matrix is of great interest to achieve reproducible and homogeneous top contacts or structural processing [12]. In this paper, we show that a combination of ultrasonic agitation, gold-chemical etching, and silicon plasma etching enables the achievement of high-density arrays of silicon nanowires with a very good length control and homogeneity on a silicon substrate. The nanowires have a good crystalline quality, and the array features good antireflective properties that could be useful for their implementations in devices such as detectors.

## Methods

**Figure 1:**
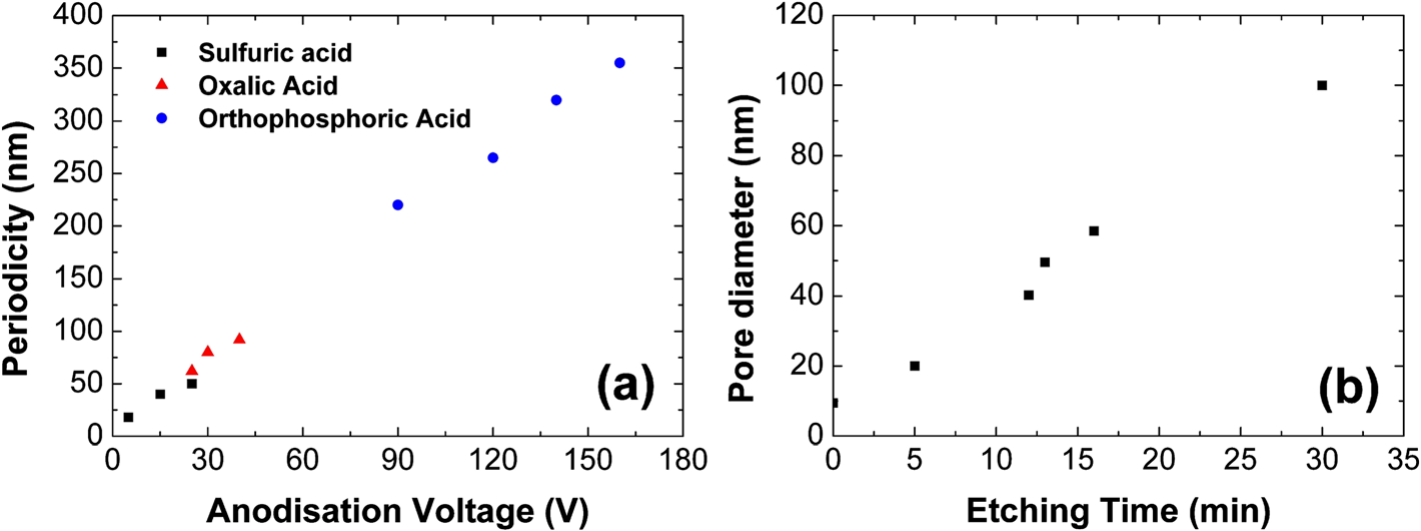
**Controlling the geometry of the AAO template.** (**a**) Periodicity of the nanopore array can be adjusted by varying the anodization voltage and the acid used. (**b**) Diameter of the nanopores is controlled by a chemical etching in phosphoric acid (7 wt.%, 30°C), the plot is for a 40-V alumina.

AAO growth templates are produced by electrochemical anodization of a thin film of aluminum deposited by plasma vapor deposition on a (100)-oriented silicon substrate. Before deposition, silicon substrates are cleaned using acetone and isopropyl alcohol (IPA). Native oxide is removed in 1% hydrofluoric acid (HF) to ensure a good electrical contact between the silicon substrate and the aluminum thin film, providing a better homogeneity during the anodization process. The initial thickness of the aluminum film has to be carefully chosen because it will determine the future length of the nanowires. Indeed, assuming the dilatation coefficient between aluminum and alumina, *a* = 1.52, the final thickness of the AAO growth template can be calculated. In our case, typical aluminum thickness available is between 1 and 10 μm leading to an alumina up to 15 μm thick. Anodization is carried out in a homemade electrochemical cell using an electrochemically active acid such as oxalic acid (C_2_H_2_O_4_). The periodicity of the nanopore array is adjusted by controlling the anodization voltage and changing the acid. It can be tuned from around 30 up to 400 nm (Figure 1a) by adjusting the voltage in the range of 10 to 200 V. To achieve a good organization of the AAO template, a double anodization process [20] can be used. The nanopores are then arranged hexagonally following the aluminum grains. Nanoimprint techniques can also be used to produce perfectly hexagonal arrays of nanopores without any perturbations from the initial structure of the aluminum film [21]. Once AAO formation is achieved, the remaining barrier layer of alumina at the bottom of the pores is removed by wet chemical etching in a solution of phosphoric acid (H_3_PO_4_) at 30°C (7 wt.% ). This etching step also allows the control of the nanopores diameter by enlarging them (Figure 1b). Gold catalyst is then deposited at the bottom of each pore using electrodeposition. A current flow is applied between the substrate and an aqueous solution of gold (III) chloride (AuCl_3_) containing Au^3+^ ions. According to the electrochemical half reaction (Equation 1), gold is deposited at the bottom of the pores on the silicon substrate. Insulating properties of alumina prevent any gold deposition on the AAO template. Native silicon oxide can also interfere with gold deposition in the nanopores by blocking the electron flow from the substrate to the electrolyte. A deoxidation using vapor HF etching is therefore undertaken before catalyst deposition to remove any traces of native oxide at the bottom of every pores of the template, thus improving gold deposition yield.

(1) $$Au_{\mathrm{aq}}^{3+}+3e^{-}\to Au_{s}$$

**Figure 2:**
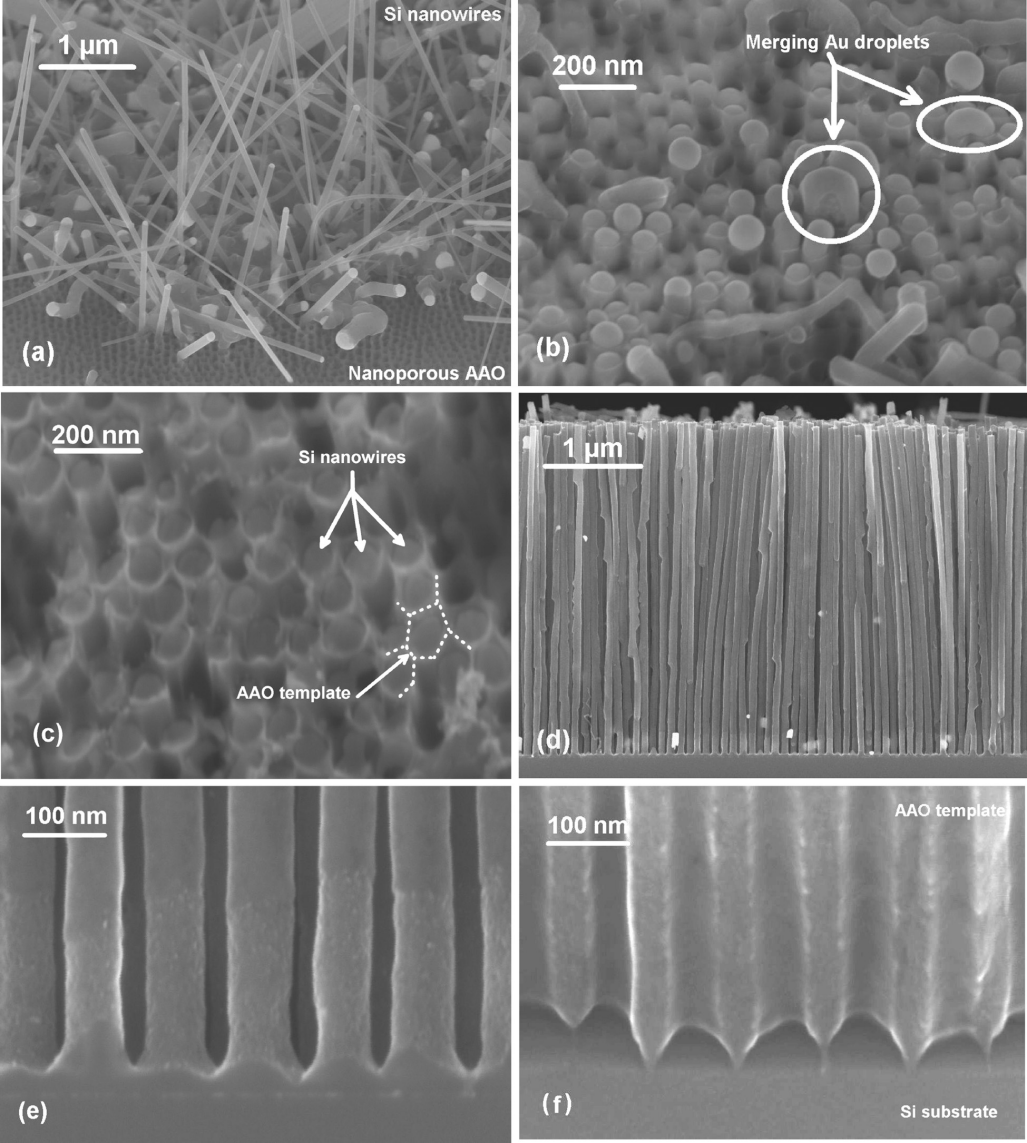
**Scanning electron microscopy image of a silicon nanowire array planarization.** (**a**) After growth, the AAO template is filled with silicon nanowires which grew out of it. (**b**) Sonication of the sample breaks the outer nanowires revealing the post-growth AAO surface. (**c**) I-KI gold etching and ICP silicon etching leads to the planarization of the nanowire array. (**d**) Cross section showing the ‘top-down like’ nanowire array with a very good homogeneity of length and high density after alumina removal by HF etching. (**e**) Close view of the interface between the Si (100) substrate and the individualized nanowires. (**f**) Empty AAO template before gold catalyst electrodeposition, complementary to the geometry of (**e**).

Subsequently, silicon nanowire growth is performed in a commercial hot-wall low-pressure CVD reactor. A flux of 50 sccm of silane (SiH_4_) carried by 1,400 sccm of hydrogen (H_2_) is injected at 580°C under a pressure of 3 Torr. It is known that these experimental conditions allow the diffusion of silane towards the bottom of the pores [19,22], therefore enabling nanowires’ growth. Addition of gaseous hydrogen chloride during growth [23] is crucial because it prevents the gold catalyst from diffusing on alumina and escaping from the nanopores, which would lead to the growth of silicon nanowires on the top of the AAO template in an uncontrolled way. Growth is carried out for 25 to 35 min depending on the AAO thickness, long enough to let the wires grow out of the template. After growth, the samples are therefore constituted of a silicon substrate with an AAO template filled with silicon nanowires. The nanowires, which grew out of the template, present neither organization nor constant diameter as can be seen on the scanning electron microscope (SEM) picture of Figure 2a. Indeed, when nanowires reach the surface of the AAO, growth conditions change abruptly leading to kinks in their growth direction. Besides, the density of circular nanopores is so high that the catalyst droplets of two or more adjacent nanowires are close enough to merge and form a bigger single droplet, leading to the growth of a larger diameter nanowire. To remove these unorganized outer nanowires, samples are sonicated for 1 min in IPA. Ultrasonic vibrations break the nanowires close to their interface with the AAO template. The surface of the nanowire array turns clean, and the only remaining structures coming out of the AAO are a few nanometers of silicon nanowires (Figure 2b). After this step, we also notice the presence of nanowires which just reached the surface of the AAO and did not grow out of it. Their catalyst droplets are at the interface with free space, sometimes merging with other ones to produce the larger diameter nanowires noticed in Figure 2a. To further clean and planarize the nanowire array, the remaining gold is removed by chemical etching for 2 min in an I-KI solution. An inductively coupled plasma (ICP) of SF_6_ and Ar is used to physically etch the exceeding silicon and separate the nanowires which began to merge. After this step, nanowires are all individualized and come up to the AAO surface (Figure 2c). The growth template is eventually etched in HF (1% aqueous solution) to free the silicon nanowire array (Figure 2d). Figure 2e shows that nanowires are well individualized with a diameter of around 70 nm following a sharp distribution. The increased roughness and conical shape at the bottom of the nanowires is reflecting the shape of the nanopores close to the interface with the substrate (Figure 2f).

Structural characterizations were carried out using a Zeiss Ultra 55 SEM (Carl Zeiss, Inc., Oberkochen, Germany) and a Jeol 3010 transmission electron microscope (TEM, JEOL Ltd., Akishima-shi, Japan). Grazing incidence X-ray diffraction (GIXD) was performed at the BM2-D2AM beamline of the European Synchrotron Radiation Facility (ESRF), Grenoble, France. Reflectivity measurements were carried out with a homemade optical setup.

## Results and discussion

SEM pictures of Figure 2 clearly show the very high density of individualized nanowires. Based on the number of nanowires counted on SEM images, we estimate the density to around 8×10^9^ nanowires cm^−2^ for a sample in which growth template was made at 40 V. It is also clear that nanowires were guided in the nanopores during their growth as revealed by the roughness of their surface: the morphology of the nanopores’ sidewalls was transferred to the growing nanowires which were thoroughly filling them (Figure 2e,f). The combination of standard microelectronics processes with the confined VLS growth of silicon nanowires therefore enabled the production of arrays of nanowires presenting similar features than with top-down techniques: their density is very high and every single nanowire is well individualized.

**Figure 3:**
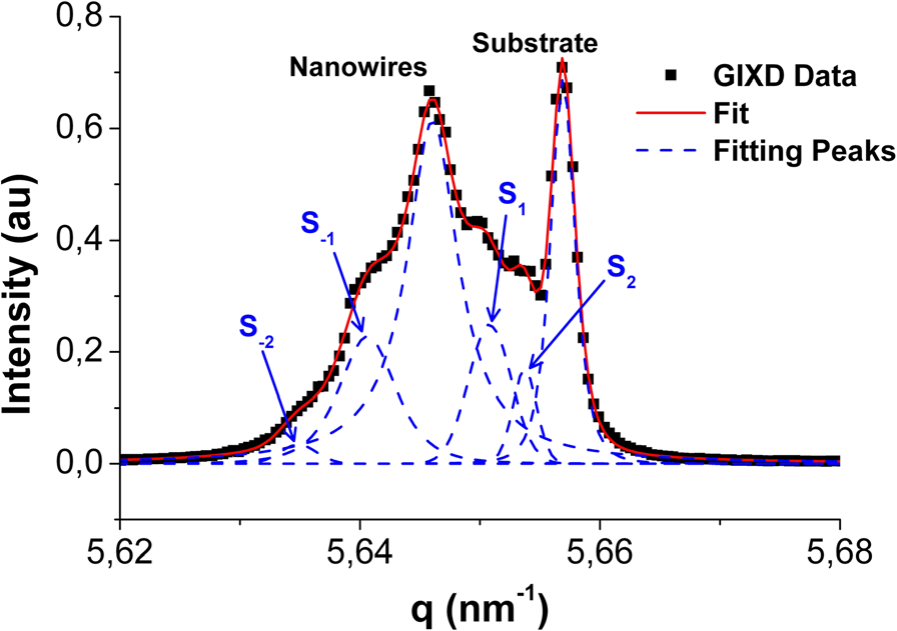
**X-ray diffraction.** Grazing incidence X-ray diffraction of a silicon nanowire array grown on a Si (100) substrate near the (−440) reflection of the substrate. The fit of the diffraction pattern reveals satellites of the nanowires’ peak (labeled S_−2_, S_−1_, S_1_, and S_2_) due to the good diameter homogeneity of the array.

GIXD on these high-density silicon nanowire arrays was performed in the light of synchrotron radiation at an energy *E* = 10.8 keV (*λ* = 0.1148 nm) in order to verify the nanowire crystalline quality and orientation. Figure 3 displays a *θ*-2*θ* diffraction pattern acquired near the (−440) reflection of the silicon substrate at *q* = 5.657 nm^−1^, with *q* as the scattering vector defined by *q* = 4*π*sin(*θ*)/*λ*. Diffraction experiments were carried out using the GIXD geometry to avoid complete overload of the signal by the substrate [24]. Two peaks are clearly visible in Figure 3, revealing the contribution of the substrate at *q* ≈ 5.657 nm^−1^ and one of the nanowires at a lower *q*. The presence of a nanowire peak ensures that the observed nanowires are crystalline and oriented in the same crystallographic direction than the substrate. Thus, the diffracting nanowires are in epitaxy with the substrate, and their crystallographic growth direction is [100] instead of the usual [111] direction. The confined growth therefore leads to silicon nanowires oriented in a different crystallographic direction than their preferential one without affecting their crystalline quality. The fit of the GIXD pattern by Pearson VII phenomenological functions shows the presence of multiple satellite peaks on both sides of the nanowires’ contribution. The presence of these satellites is due to the constant diameter of the nanowires within the array. Based on the angular distance Δ*ω* between the satellites and the nanowires peak [25,26], it is possible to compute the diameter *D* of the nanowires using Equation 2.

(2) $$D=\frac{n.\lambda .\cos\left(\theta \right)}{\Delta \omega .\sin\left(2\theta \right)}$$

with *n* as the order of the satellite peak, *λ* as the X-ray beam wavelength, and *θ* as the Bragg angle. The calculated diameter is *D* = 69 nm which is consistent with the measurements made on SEM pictures at the scale of a few nanowires such as Figure 2e. However, the dimensions extracted from the results of X-ray diffraction are averaged on the whole sample and are then giving evidence that the array is homogeneous on the full sample. The GIXD measurements also highlight the presence of a mechanical strain in the diffracting nanowires revealed by the difference in the scattering vector of the nanowire and substrate peaks. The lattice parameter mismatch expressed as Δ*a*/*a* = (*a*_SiNWs_−*a*_Sub_) / *a*_Sub_ can indeed be related to the shift of the scattering vector using Bragg’s law 2*d*sin(*θ*) = m*λ* and the definition of the scattering vector *q*:

(3) $$\frac{\mathrm{\Delta a}}{a}=\frac{q_{\text{Substrate}}-q_{\text{NWs}}}{q_{\text{NWs}}}$$

Since the nanowires’ diffraction peak appears at a lower scattering vector than the substrate one, the silicon lattice parameter is slightly dilated in the nanowires compared to bulk silicon. The calculated strain using Equation 3 is *Δa*/*a* = 1.9 × 10^− 3^ which is one order of magnitude greater than for gold-catalyzed silicon nanowires which grew freely [24]. This increased strain could be explained by the forced growth in the nonpreferential [100] crystallographic direction or by the effects at the interface between the growing nanowires and their Al_2_O_3_ growth template, but this still needs further investigation.

**Figure 4:**
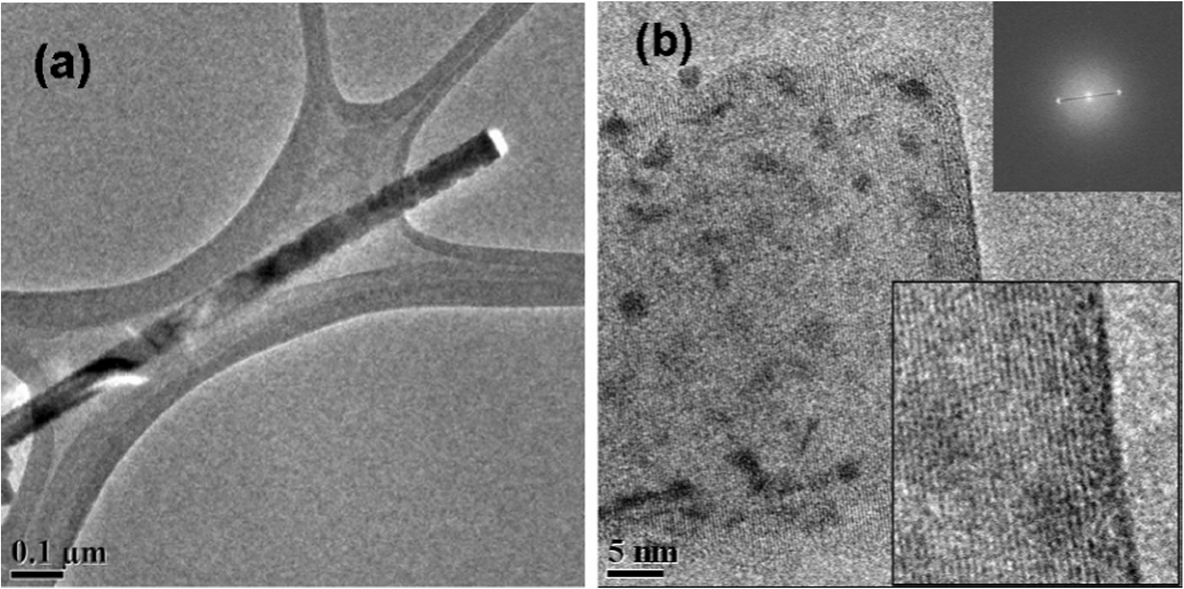
**Transmission electron microscopy.** TEM view of a silicon nanowire which grew in the AAO template. (**a**) Low-resolution view of the nanowire. (**b**) High-resolution picture near the apex of the nanowire. Upper inset is an FFT of the image showing the periodicity along the growth axis corresponding to the (111) planes of silicon. Lower inset presents a high-resolution view clearly displaying the (111) planes.

Structural investigations were also carried out using TEM on eight different single nanowires taken from two samples. Figure 4a displays a TEM image of a whole nanowire, while Figure 4b shows a high-resolution picture of the nanowire revealing its silicon lattice. No defects were detected in the crystalline matrix of any of the observed nanowires which give evidence of their very good crystallinity. Fast Fourier transform (FFT) of TEM pictures (inset of Figure 4b) of all observed nanowires show that the (111) planes of silicon are oriented perpendicular to the growth axis. The observed nanowires therefore grew along the [111] direction, which is different from the ones characterized by GIXD and from the substrate orientation (100). In this case, there is no epitaxial relation between the nanowires and their substrate. The monocrystalline quality of the observed [111] nanowires in spite of their nonepitaxial growth is an important feature for the possible future use of this technique on noncrystalline substrates such as stainless steel or glass. It ensures that semiconductor nanowires can be grown on universal substrates with a very good crystalline quality. We also notice on the TEM pictures that the nanowires’ surface presents low-contrast clusters. Energy dispersive X-ray microanalysis of these areas did not allow any detection of contamination materials such as aluminum (unshown results). This feature could be actually caused by topography effects due to the roughness of the nanowires’ surface as described in Figure 2e.

Two types of nanowires therefore grew in the AAO template, one in epitaxy with the (100) substrate and another one with no crystalline relation with it, each type being clearly detected with a separate technique. Using SEM pictures such as the one of Figure 2e, it is not possible to visually differentiate between the two types of wires since they are all well individualized and fully guided in the nanopores. The most likely cause for the nonepitaxial nanowire growth is a partial deoxidation of the silicon substrate during the vapor HF step before catalyst electrodeposition. If the silicon surface at the bottom of a pore is only partially deoxidized, the remaining native oxide would disturb the initial growth steps by screening the substrate and therefore preventing a good epitaxy. This effect is known and described in the case of copper electrodeposition in nanoporous alumina [27]. In the opposite case of a thorough deoxidation of the nanopore, the resulting nanowires would grow in epitaxy with the silicon substrate.

**Figure 5:**
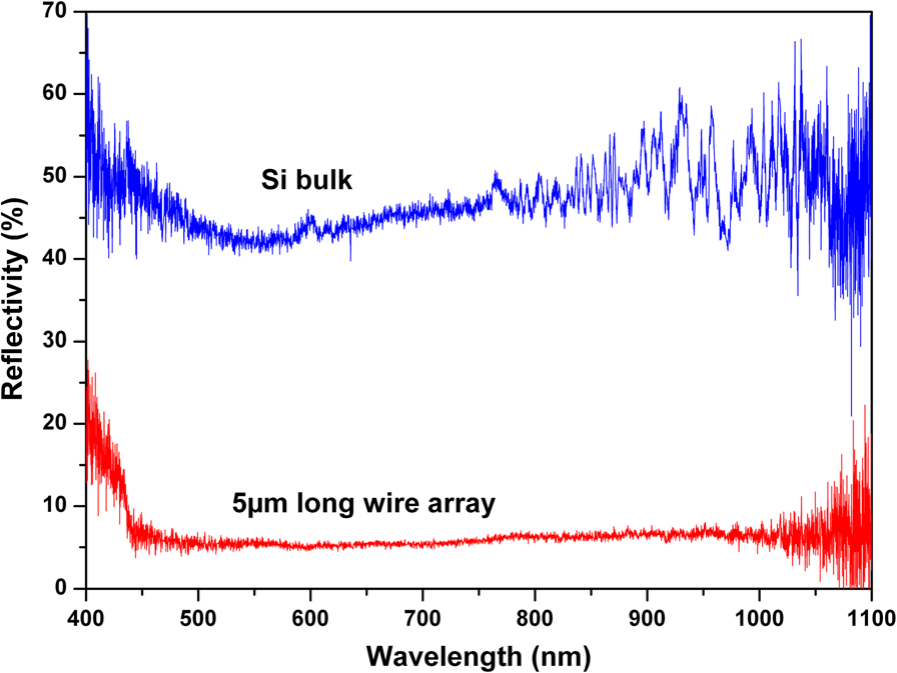
**Reflectivity.** Measured reflection coefficient for bulk silicon (blue) and a 5-μm-long silicon nanowire array (red).

The reflectivity of the ultradense silicon nanowire arrays was also characterized to verify the effectiveness of light trapping in the structure as predicted by simulations [28,29]. Reflectivity measurement on a 5-μm-long silicon nanowire array is presented in Figure 5 and shows a strong difference compared to bulk silicon. Reflectivity is indeed reduced from 45 to around 5%, revealing a strong absorption of light by the nanostructured surface of the sample. It is interesting to notice that even if the nanowires are not as perfectly ordered as in simulations or with lithographically patterned top-down arrays, light absorption is still greatly improved close to 1. This enhanced optical property combined with the very high density of nanowires on the samples is very promising towards the future use of this kind of nanowire arrays as detectors or photovoltaic devices.

## Conclusions

Silicon nanowire arrays were produced presenting top-down features but using a bottom-up CVD process. A very high density was reached with a planarized overall surface and long-range periodicity leading to interesting optical behavior such as an increased light absorption. Silicon nanowires are monocrystalline and grew on a nonpreferential (100) silicon substrate, opening the way to the use of this technique on noncrystalline universal substrates such as glass or metals.

## Competing interests

The authors declare that they have no competing interests.

## Authors’ contributions

LD wrote the paper, performed scanning electron microscopy, and optical measurements. LD, TG, and TB developed and characterized the alumina template. LD and PG grew the nanowires. LD, TG, HR, and DB carried out the diffraction experiments. AL made the transmission electron microscope pictures and analysis. All authors read and approved the final manuscript.
